# Steady State Vapor Bubble in Pool Boiling

**DOI:** 10.1038/srep20240

**Published:** 2016-02-03

**Authors:** An Zou, Ashish Chanana, Amit Agrawal, Peter C. Wayner, Shalabh C. Maroo

**Affiliations:** 1 Department of Mechanical & Aerospace Engineering, Syracuse University, Syracuse NY 13244 USA; 2 Department of Electrical Engineering and Computer Science, Syracuse University, Syracuse NY 13244 USA; 3 Center for Nanoscale Science and Technology, National Institute of Standards and Technology, Gaithersburg MD 20899 USA; 4 Maryland Nanocenter, University of Maryland, College Park MD 20742 USA; 5 Department of Chemical & Biological Engineering, Rensselaer Polytechnic Institute, Troy NY 12180 USA

## Abstract

Boiling, a dynamic and multiscale process, has been studied for several decades; however, a comprehensive understanding of the process is still lacking. The bubble ebullition cycle, which occurs over millisecond time-span, makes it extremely challenging to study near-surface interfacial characteristics of a single bubble. Here, we create a steady-state vapor bubble that can remain stable for hours in a pool of sub-cooled water using a femtosecond laser source. The stability of the bubble allows us to measure the contact-angle and perform *in-situ* imaging of the contact-line region and the microlayer, on hydrophilic and hydrophobic surfaces and in both degassed and regular (with dissolved air) water. The early growth stage of vapor bubble in degassed water shows a completely wetted bubble base with the microlayer, and the bubble does not depart from the surface due to reduced liquid pressure in the microlayer. Using experimental data and numerical simulations, we obtain permissible range of maximum heat transfer coefficient possible in nucleate boiling and the width of the evaporating layer in the contact-line region. This technique of creating and measuring fundamental characteristics of a stable vapor bubble will facilitate rational design of nanostructures for boiling enhancement and advance thermal management in electronics.

Boiling is one of the most efficient heat transfer mechanisms that allows a large amount of heat to be transferred over small surface areas due to the associated phase-change process. It has been widely used in industry^1^, from cooling small electronics to large power plants. However, it is a complex phenomenon which involves multiple length scales present at the base of a bubble in the contact line region^2^,^3^. The three-phase contact line region (Fig. 1a), where the liquid-vapor interface meets the solid surface, can be divided into three sub-regions of varying thicknesses: non-evaporating film region (of nanometer-scale thickness), evaporating film region (of micrometer-scale thickness), and bulk meniscus region (of micrometer- to millimeter-scale thickness)^4^,^5^, with these regions constituting the microlayer. Contact line models^6^,^7^, together with transient conduction^8^ and microlayer evaporation^9^,^10^, have been widely accepted as the basic heat-transfer mechanisms in boiling. The dynamics of contact line region and the microlayer dictate bubble growth and departure, and are of significant importance in understanding the fundamental behavior of the boiling phenomenon^11^. Visualization of the boiling process and the contact line region has recently been pursued^12^,^13^,^14^,^15^,^16^,^17^ with tremendous impact in providing a realistic depiction of the boiling process; however, the unsteady nature and a short time-span of the bubble ebullition cycle has made *in-situ* imaging of a single bubble very challenging.

The contact line region is incorporated into predictive boiling models through contact angle values^18^,^19^,^20^. The intricacies involving the shape of an interface and the behavior of the contact line are implicitly accounted for in the contact angle, thus making the bubble contact angle parameter of significant importance in boiling models. Methods that have been applied to determine the contact angle include the captive-bubble technique (involves an air-bubble)^21^,^22^,^23^, the flotometric technique (involves solid particles interacting with a bubble)^24^, and high-speed photography (involves visual approximation of the contact line region and angle)^25^. In addition, equilibrium or advancing contact angles of a liquid droplet on the surface at room temperature are often used boiling models^19^,^26^, although it is difficult to relate the boiling process to the droplet wetting characteristics due to the highly transient conditions associated with liquid-vapor phase change^27^,^28^,^29^. Thus, the contact angle of a vapor bubble in pool boiling has yet to be measured in the early growth stages due to the dynamic nature of the bubble ebullition cycle; making it all the more necessary to image the contact line region to advance our understanding of boiling process and enhance boiling heat transfer efficiency.

In this work, we create a steady-state vapor bubble in a pool of sub-cooled water by heating the surface with a femtosecond laser source. The bubble remains stationary for hours, allowing *in-situ* imaging of the microlayer and the contact line region, as well as measurements of the contact angle. A Ti:Sapphire ultrafast laser (pulse length ≈120 fs, repetition rate = 80 MHz, center wavelength *λ*_0_ = 800 nm) in conjunction with a second-harmonic generation (SHG) unit was used to generate high-power laser pulses at a free-space wavelength of *λ*_SHG_ = 400 nm. The laser pulses were passed through a 5× or 50× objective lens and focused on an absorbing 40 nm thick Au film that was sandwiched between a silica glass substrate (bottom-surface) and sputter-deposited 400 nm thick layer of SiO_2_ (top-surface). The focused laser beam creates a highly localized heating area corresponding to the beam-diameter ≈170 μm. To achieve boiling, a pool of water was created inside a 6 cm long and 1.4 cm inner diameter glass tube bonded to the SiO_2_ top-surface of the substrate. Although laser-initiated bubbles have been used in literature^30^,^31^,^32^, fundamental characteristics of formation of such bubbles or their *in-situ* imaging to understand the boiling process have not been explored. Experiments were performed in both regular deionized (DI) water with dissolved air and degassed DI water. The latter was prepared by boiling regular DI water for one-hour, and filling it inside the glass-tube using a 220 nm filter syringe to remove any particulates. Based on the one-dimensional (1D) diffusion equation, it would take >24 hours for the atmospheric air to diffuse to the bottom of the 6 cm long tube whereas each experimental measurements lasted for <2 hours. Convection currents due to bubble formation can increase diffusion of air in water; however, experimental observations show that water remained degassed near the surface as the vapor bubble condensed when the laser was turned off (video V1 in the supplementary material). Using regular DI water, we tested hydrophilic SiO_2_ surfaces (where experiments were performed immediately after plasma cleaning) and normal SiO_2_ surfaces. Similarly, using degassed DI water, we tested both the normal SiO_2_ surface and a hydrophobic tridecafluoro−1,1,2,2-tetrahydrooctyl-trichlorosilane (FOTS) surface. In Table 1, we list the drop contact angle on these surfaces measured using a goniometer (drop images in supplementary information). After a bubble is formed on the surface through heating with a laser pulse, we increase the average power of the laser by 20 mW for every subsequent reading of the bubble base, bubble diameter and contact angle. The measurements were stopped before the average laser power could reach the damage threshold of the SiO_2_ surface (corresponding to an average power ≈240 mW). The uncertainty in all the contact angle measurements are one standard deviation for repeated experimental measurements (five in total). Please refer to the supplementary information for details on the experimental setup, sample fabrication and preparation.

Figure 2a–c show the bottom view, obtained using an inverted optical microscope, of a single bubble formed on hydrophilic SiO_2_, FOTS and normal SiO_2_ surfaces, respectively, with increasing laser heating power. The vapor bubble instantly achieves steady-state in degassed DI water as the heat transfer from the surface leads to continuous evaporation of water in the microlayer which is balanced by the continuous condensation of vapor at the liquid-vapor interface away from the surface due to the sub-cooled pool of water (temperature of water was ≈75 °C lower than the saturation temperature ≈100 °C). The bubble contact angle 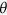 for the hydrophilic SiO_2_ surface was obtained by measuring the bubble base diameter, 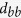, and the height of bubble middle plane, 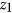, (Fig. 2d1) and determined by the equation: 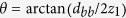; whereas for the hydrophobic surface (FOTS), 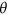 was obtained by the first-order derivative of the parabolic curve of the interface (Fig. 2d2) and given by 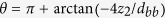 where the height of the bubble is 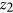. In these equations, the bubble base diameter, 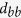, was obtained directly from the calibrated optical images acquired using a CCD camera; while the *z* positions of the bubble middle plane (for hydrophilic and normal SiO_2_) or the bubble height (for the FOTS surface) were obtained by translating the focal plane of the objective to the appropriate *z* height and reading the *z*-offset from the controller of the motorized translation stage. Figure 2e,f show the variation in the bubble base/bubble diameter and contact angle, respectively, with increasing laser power for the various cases studied. The bubble sizes were consistently smaller in degassed DI (D) water when compared to regular DI (R) water due to the contribution of dissolved air in the bubble growth phenomenon in regular water. This effect was further confirmed by turning the laser off; the bubble in degassed DI water disappeared in < 20 seconds (due to condensation of vapor) while the bubble in regular DI water decreased slightly in diameter but stayed on the surface for days (videos V1, V2 and V3 in the supplementary material). Our results are also consistent with a recent study^33^ where air nanobubbles were found to be stable for days due to the slow-rate of dissolution of air into an already saturated surrounding liquid.

For the normal SiO_2_ surface with degassed water, the bubble contact angle decreased with increasing laser power (from 73.6° ± 3.9° at 120 mW to 45.3° ± 5.2° at 200 mW). In all the other cases, the bubble contact angle was found to be independent of the laser power studied. Average bubble contact angle in regular water was determined to be 31.9° ± 0.5° on normal SiO_2_ surface, which is similar to the drop contact angle (Table 1), and in good agreement^26^ with the drop receding contact angle after boiling experiments (32.3° ± 0.4°). The average contact angle was 29.3° ± 0.4° on the hydrophilic SiO_2_ surface which is slightly smaller than the bubble contact angle on normal SiO_2_ surface. Contact angle of the bubble on FOTS surface was 96.8° ± 0.2° which is also similar to the measured drop contact angle. These variations in contact angle, especially between degassed and regular water on the same surface, depend on the dynamics of the microlayer and contact line region as studied and explained below.

A stable bubble enables *in-situ* imaging of the contact line region present at the base of the bubble. Using femtosecond laser illumination through a 50× microscope objective, bubbles were formed on a normal SiO_2_ (Fig. 3a) surface with regular water, a normal SiO_2_ surface with degassed water (Fig. 3b), and a FOTS surface with degassed water (Fig. 3c). The contact line region is imaged (Fig. 3) using the inverted optical microscope under illumination from both a white halogen lamp source (Fig. 3a1) and a 632 nm HeNe laser (Fig. 3a2). The microlayer and the contact line region were identified based on the juxtaposition of these two sets of images. Under coherent HeNe laser illumination, two sets of fringes were observed in the images that are a result of thin-film interference associated with interaction between regions of different refractive index. The first set of fringes, F-1 (dark thick partial rings), have fringe-gaps decreasing in the outward radial direction and are associated with interference resulting from the top curved interface of the bubble (and not due to the contact line region). The second set of fringes, F-2, are relatively closely packed and the fringe-gap for these set of fringes increase in the outward radial direction. These fringes are a result of thin-film interference of incident light with the partially reflected light within the thin liquid microlayer present at the base of the bubble^14^, with the increase in fringe-gap attributed to the increase in radius-of-curvature of the microlayer in the outward radial direction. The second set of fringes is clearly evident in degassed DI water (Fig. 3b2) showing the presence of a liquid microlayer over the entire bubble base. However, in regular DI water (Fig. 3a2), the fringes are absent from the center of the bubble base and are only present in the equivalent bright regions of Fig. 3a1. This observation implies the presence of a dry-spot region at the center of the bubble base and the formation of the three-phase contact line region (liquid-vapor-solid) interfacing with the SiO_2_ surface, with a significantly reduced microlayer. The microlayer shape obtained for a bubble on normal SiO_2_ in regular water and degassed water, and on FOTS surface in degassed water is plotted in Fig. 2a3 respectively. As the interference of the monochromatic light source generates dark and bright fringes corresponding to constructive and destructive interference respectively, these fringes are separated by an optical path difference equal to effective half wavelength, *nλ*_0_/2, where *n* is the refractive of the medium, *λ*_0_ is the free-space wavelength of light. The position of these fringes is used to construct the shape of the microlayer, where the difference in local thickness at the adjacent bright/dark fringe location *t*_*m* + *1*_ and *t*_*m*_ is given by *t*_*m* + *1*_ – *t*_*m*_ = *λ*_0_/2*n* cos(*θ*) for the light refracted at angle *θ* into the microlayer.

The fringes observed in the contact line region are also used to explain the experimentally measured contact angle values. In the regular DI water on normal SiO_2_ surface, the larger size of the bubble (due to contribution of dissolved air) at low laser power creates a dry spot at the center causing the creation of a three-phase contact line; and hence, the bubble contact angle is similar to the drop contact angle (where a similar three-phase contact line is present). However, with degassed DI water on normal SiO_2_ surface, the microlayer covers the entire bubble base preventing the formation of the three-phase contact line, and the contact angle is governed by the microlayer curvature relative to the bubble curvature. Hence, the contact angle decreases with increasing laser power as the radius of curvature of the microlayer increases significantly faster compared to the radius of curvature of the bubble. Similarly in FOTS, the larger radius of curvature microlayer along with the parabolic bubble shape results in large contact angle values. The parabolic shape of bubble is attributed to the larger bubble base diameter as the reduced wettability of the hydrophobic surface requires a larger microlayer to remove the same amount of heat from the surface. However, for degassed DI water on both normal SiO_2_ and FOTS surfaces, it is expected that after a critical bubble size is reached – the microlayer would reduce in thickness, form the three-phase contact line and the bubble contact angle would converge to that of the drop contact angle.Further, bubbles with the microlayer wetting the entire bubble base will not depart the surface as the capillary and disjoining suction force (due to reduced liquid pressure in microlayer) is estimated to be larger than the buoyancy force and capillary force at the top of the bubble (please refer to supplementary information for detailed analysis).

Maximum heat flux occurs in the thin evaporating region^10^ and is of critical importance in bubble growth dynamics; however, knowledge of heat transfer coefficient and corresponding width of this region is currently lacking in literature. We use experimental data from *in-situ* imaging of the contact line region together with finite-element-method based numerical simulations to characterize the evaporating region in the microlayer. We first focus on the bubble formation in regular DI water on normal SiO_2_ surface to obtain experimental data. Interestingly, it was found that the bubble grew gradually at constant laser power. The source of bubble growth results from the air dissolved in the water, which is released into the bubble during the vaporization of water from the evaporating region of the microlayer. The bubble grew steadily (Fig. 4a) at a volumetric rate of (5.60 ± 0.06) × 10^−3^ mm^3^/min (please refer to supplementary information for bubble volume calculations) and the contact line region at the bubble base grew radially outward at a speed of (1.9 ± 0.1) μm/min during the initial 40 min, but stopped after it reached a diameter of ≈270 μm (Fig. 4b); this limiting diameter approximately corresponds to the measured laser beam diameter (≈170 μm) with additional radial heat conduction in the Au layer. The uncertainty in the measurements of the bubble growth rate and bubble base are standard deviation of the fit parameter. Contact angle of the bubble (Fig. 4c) decreased with time as the bubble base remained nearly constant while the bubble diameter grew uninhibited. Similar to Fig. 3a1, the central dry spot diameter was identified from *in-situ* imaging of the contact line region. Based on these experimental data, the heat transfer rate *q* in the evaporating region, 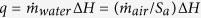, is obtained from the air-water solubility mass balance calculation. Here, 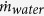 is the vaporization rate of water in evaporating region, 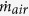 is the mass flow rate of air into the vapor bubble from evaporating region, *S*_*a*_ is the solubility of air in water, and Δ*H* is the latent heat of vaporization.

The heat transfer rate in the evaporating region *q* is also dependent on the overall heat transfer coefficient *h* and the area of the evaporating region through (please refer to Supplementary Information): 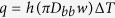 where *D*_*bb*_ is the central dry spot diameter, *w* is the width of evaporating region, and Δ*T* is the temperature difference between the surface and the bulk fluid. Unknown parameters *h* and *w* characterize the evaporating region (Fig. 4d), and we performed finite-element-method based simulations to determine the range of *h* and *w* for which the simulated release rate of air from the evaporating region agreed with that obtained through measured bubble geometry in the experiments (Fig. 4a). An axi-symmetric domain was considered that included the glass substrate, 40 nm Au layer and 400 nm SiO_2_ layer (Fig. 4d). A parametric study was performed where *h* and *w* were varied from 5000 Wm^−2^K^−1^ to 200,000 Wm^−2^K^−1^, and from 0.5 μm to 19.5 μm, respectively for a total of 3500 simulation cases. Figure 3e shows the range of *h* and *w* for which simulation results were in good agreement with experiments within a standard uncertainty of 4.5%. The temperature profile of the surface is plotted for this range (Fig. 4f). Interestingly, the surface temperature at *r *≈* *135 μm was ≈39 °C, which is the critical temperature when Marangoni flow inhibits fluid flow towards the contact line^34^, thus equilibrating the incoming mass flow to the evaporation rate and causing the contact line to become stable at bubble base diameter of ≈270 μm. The temperature at the center of the bubble is calculated to be ≈82 °C, which is also in good agreement with experiments^35^, where it has been shown that the formation of a bubble in pool boiling in sub-cooled water at room temperature occurs at ≈84 °C. The thermal boundary layer thickness prior to bubble nucleation is simulated to be ≈200 μm, and around the steady bubble is estimated to be ≈280 μm for *h* = 120 kW/m^2^K and *w* = 10 μm (please refer to Supplementary Information).

In summary, a steady-state vapor bubble is created in a pool of sub-cooled water by femtosecond laser heating which allows for *in-situ* imaging of the microlayer and the contact line region. The bubble can remain stable for hours as the evaporation of water at the surface is balanced by condensation of vapor at the liquid-vapor interface inside the bubble. Experiments are conducted on hydrophilic (SiO_2_) and hydrophobic (FOTS) surfaces in regular (with dissolved air) and degassed DI water. The contact angle of the vapor bubble is measured for various cases, and the microlayer and contact line region are imaged with white light and a coherent laser source. For the laser powers studied, it was found that the three-phase contact line readily forms in regular DI water, while the microlayer covers the entire bubble base in degassed DI water. The contact angle for the bubble is found to resemble the drop contact angle on the same surface if the three-phase contact line forms, otherwise the contact angle is dependent on the curvature of the microlayer and the bubble, and decreases with increasing laser power. The evaporating region in the contact line region is characterized by numerical simulations and experimental results, and permissible values of heat transfer coefficient and corresponding width are calculated, thus providing an estimate to the upper limit of the heat transfer coefficient attainable in nucleate boiling as well as thin-film evaporation. The work presented here will advance the design of nanostructures to enhance heat transfer by optimizing the width of microlayer and improve our understanding of boiling phenomenon, particularly in outer-space where lack of gravity causes the bubbles to stay stationary on a heated surface. *In-situ* imaging of the microlayer and contact line region in a steady state bubble is a powerful technique for understanding the physical dynamics of the bubble growth process.

## Additional Information

**How to cite this article**: Zou, A. *et al*. Steady State Vapor Bubble in Pool Boiling. *Sci. Rep*. **6**, 20240; doi: 10.1038/srep20240 (2016).

## Supplementary Material

Supplementary Information

Supplementary Movie S1

Supplementary Movie S2

Supplementary Movie S3

## Figures and Tables

**Figure 1:**
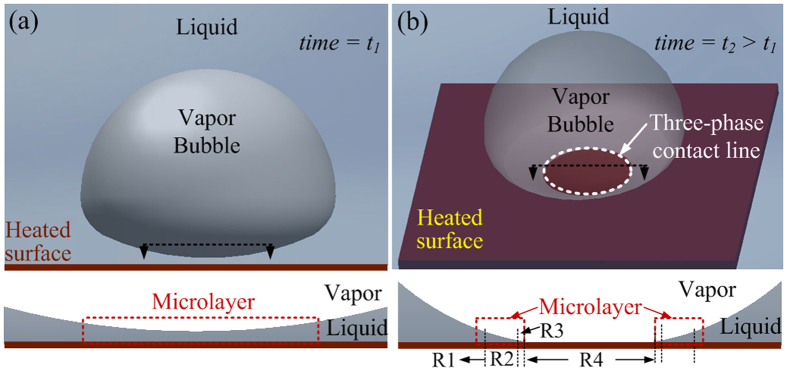
Microlayer and three-phase contact line evolution during early stages of vapor bubble growth process. (**a**) 2D schematic of vapor bubble on a heated surface with a microlayer wetting the entire bubble base (three-phase contact line is not present). Cross-section of the base of the bubble depicting the microlayer. Increase in heating temperature and bubble size forms a three-phase contact line with reduced microlayer, *i.e*., from 1a to 1b.(**b**) Three-dimensional (3D) schematic of a vapor bubble on a heated surface in a pool of liquid depicting the presence of the three-phase contact line. Cross-sectional two-dimensional (2D) view of the three-phase contact line showing the non-evaporating region (R3), evaporating film region (R2), bulk meniscus region (R1) and the dry spot (R4); microlayer comprises of regions R1, R2 and R3.

**Figure 2:**
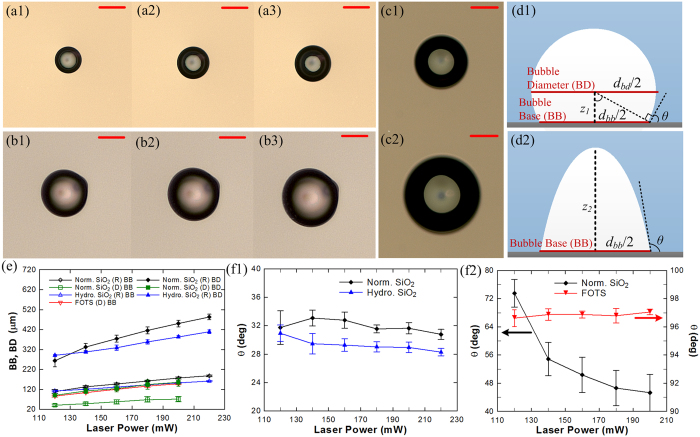
Laser heated steady-state bubble under 5× magnification captured using an inverted optical microscope on: (**a**) normal SiO_2_ surface in degassed water, (**b**) FOTS surface in degassed water, and (**c**) normal SiO_2_ surface in regular water, as a function of increasing laser power: 120 mW (**a1,b1**), 140 mW (**c1**), 160 mW (**a2,b2**) and 200 mW (**a3,b3,c2**). The bubble is twice as large for regular water when compared to the degassed water due to contribution of dissolved air; scale bars in (**a–c**) are 50 μm. (**d**) Contact angle measurement depiction on (**d1**) SiO_2_ surface (θ = arctan(*d*_*bb*_/2*z*)) and (**d2**) hydrophobic FOTS surface (θ = *π* + arctan(−4*z*/*d*_*bb*_)). (**e**) Bubble base (BB) and bubble diameter (BD) measurements on normal SiO_2_ (Norm. SiO_2_) surface in degassed (D) and regular (R) water, hydrophilic SiO_2_ (Hydro. SiO_2_) in regular water, and hydrophobic FOTS in degassed water. (**f)** Contact angles for bubbles in (**f1**) regular water and (**f2**) degassed water with increasing laser power. Bubble contact angles in regular water were found to be independent of laser power and similar to drop contact angles (shown in inset in (**f1**)), but noticeably larger in degassed water on normal SiO_2_ while dropping significantly with increase in laser power. Uncertainties in (**e**) are based on standard deviation of five measurements for every experimental data point. Uncertainties in (**f1**,**f2**) are one standard deviation based on propagation of uncertainty from five individual measurements of bubble base and *z*_*1*_ or *z*_*2*_ position for each data point. Lines connecting the data points in (**e**), (**f1**,**f2**) are guides to the eye.

**Figure 3:**
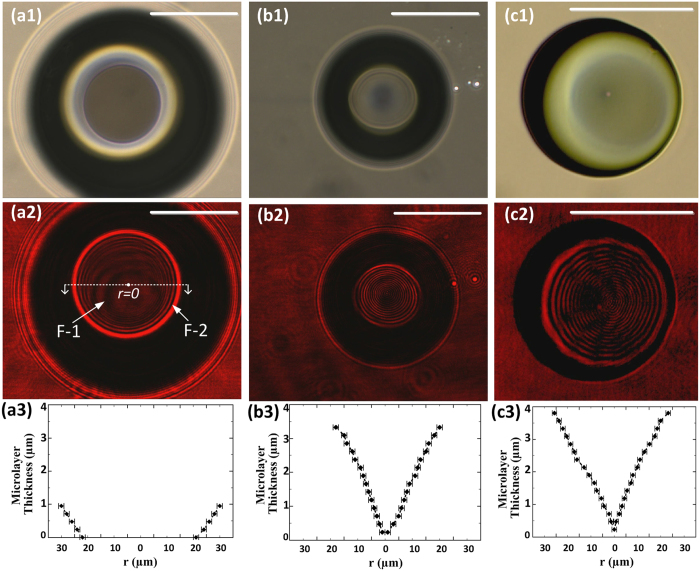
Optical images of the bubble with 50× magnification under white-light and HeNe laser illumination. (**a**) Bubble on normal SiO_2_ surface in regular water shows fringes (F-2) only in the corresponding white/bright region but absent at the center implying a central dry-spot and the narrow microlayer forming a three-phase contact line. (**b**) Bubble on normal SiO_2_ surface and (**c**) FOTS surface in degassed water, show the bubble base is completely covered with the microlayer in bubble base region and no three-phase interfacial line is formed yet. The formation of the three-phase line is expected to change the bubble contact angle to be similar to that of a drop. Secondary fringes F-1 (darker and thicker partial rings) are caused due to the interference of light with bubble curvature as light is incident from the top of the sample. The scale bar in all images is 50 μm. (**a3**,**b3**,**c3**) show the microlayer curvature (shape) obtained from the respective fringes using the thin-film interference equation. (**b3**,**c3**) do not have a dry spot and the microlayer thickness at *r *=* *0 has been assumed to emphasize this fact. Uncertainties in (**a3**,**b3**,**c3**) for radial distance *r* are determined from the smallest fringe width that can be measured based on the resolution of the camera.

**Figure 4:**
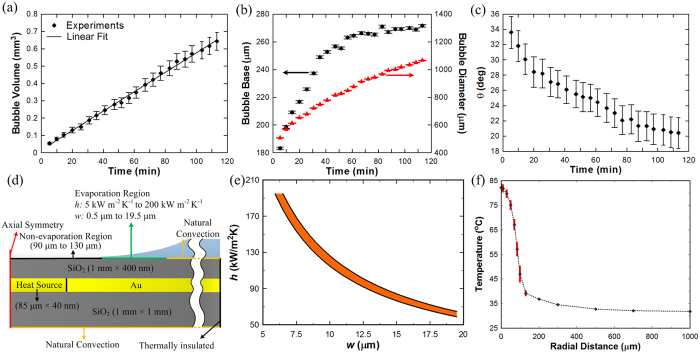
Bubble growth on normal SiO_2_ surface in regular water at constant laser power and corresponding simulation results. (**a**) Bubble volume growth rate. (**b**) Bubble base (black) and bubble diameter (red) change with time during growth; for the bubble diameter, the upper and lower limits of the error bar are too close and they merge together. (**c**) Bubble contact angle change with time during growth. (**d**) Finite-element-method based simulation domain and boundary conditions. The heat conduction inside the sample was simulated with the 2D axial symmetry condition to estimate the bubble growth rate. A large enough domain (1 mm × 1 mm) was simulated so that the right side boundary condition could be set as thermally insulated. The heat source is the gold layer which absorbs the laser (beam radius ≈ 85 μm). The heat transfer coefficient *h* and width *w* of the evaporating layer were varied in the simulations, and results for the bubble growth were compared to experimental results, within an error of 4.5%, to estimate the range of *h* and *w*, as depicted in (**e**). (**f**) Temperature profile on solid surface from simulations depicting the temperature to be ≈39 °C at bubble base radius of ≈135 μm. Uncertainties in (**a**,**c**) are based on propagation of uncertainty from five individual measurements or readings of bubble diameter and contact angle, and bubble base and *z*_*1*_/*z*_*2*_ position, respectively, for every experimental data point. Uncertainties in (**b**) are based on one standard deviation of five measurements for each data point. Rectangular red colored bars in (**f**) depict the spread in surface temperature for a subset of simulations where bubble growth rate was calculated to be within an error of 0.2% from experimental results; average temperature values are also shown and connected as a guide to the eye.

**Table 1:** Sample surfaces and liquids used for the experiments along with the static droplet contact angle on these surfaces.

Sample Surface	Drop Contact Angle	Liquid Tested
Hydrophilic SiO_2_	0°	regular DI water
Normal SiO_2_	33.4^o^ ± 2.7°	regular & degassed DI water
Trichlorosilane (FOTS)	109.8^o^ ± 2.9°	degassed DI water

Hydrophilic SiO_2_ surface was created by oxygen plasma cleaning of normal SiO_2_ surface, and used in experiments immediately afterwards. The normal SiO_2_ surface was used approx. 3 to 4 days after oxygen plasma cleaning. Uncertainties in drop contact angle are one standard deviation based on propagation of uncertainty from five individual measurements.
