# Supplementary material for: Steady State Vapor Bubble in Pool Boiling
Source: Sci Rep. 2016 Feb 3;6:20240. doi: 10.1038/srep20240 (PMC4738301; doi:10.1038/srep20240)
Supplement: Supplementary Information [file srep20240-s1.pdf]

Supplementary Information: Steady State Vapor Bubble in Pool Boiling

An Zou[1], Ashish Chanana[2], Amit Agrawal[3,4], Peter C. Wayner, Jr.[5] & Shalabh C. Maroo[1]*

[1]Department of Mechanical & Aerospace Engineering, Syracuse University, Syracuse NY 13244 USA
[2]Department of Electrical Engineering and Computer Science, Syracuse University, Syracuse NY 13244 USA
[3]Center for Nanoscale Sciences and Technology, National Institute of Standards and Technology, Gaithersburg MD 20899 USA
[4]Maryland Nanocenter, University of Maryland, College Park MD 20742 USA
[5]Department of Chemical & Biological Engineering, Rensselaer Polytechnic Institute, Troy NY 12180 USA
*Corresponding author: scmaroo@syr.edu, Phone: 315-443-2107

S1. Sample Fabrication

Figure S1 shows the cross-sectional schematic of the fabricated samples. A 10 nm thick layer of Cr, followed by a 40 nm thick Au layer and another 10 nm thick Cr layer were thermally deposited onto a silica substrate. The thin metallic layers were used to locally absorb the focused laser beam to create bubbles. A final sputter deposition of 400 nm thick layer of SiO_2 served as the hydrophilic (drop contact angle of 0° immediately after oxygen plasma cleaning) or the normal (drop contact angle recovered to 33.4° ± 2.7° few days after plasma cleaning) SiO_2 surface (Fig. S1a). The FOTS samples were fabricated by molecular vapor deposition of a single monolayer of FOTS on the SiO_2 surface (Fig. S1b).

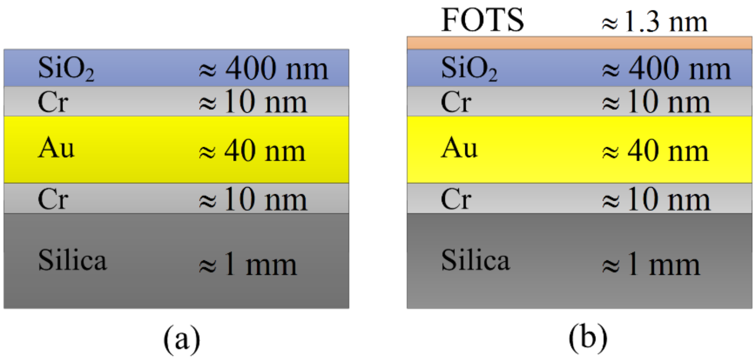

Figure S1: Cross-sectional schematic of the fabricated samples: (a) top SiO_2 surface (b) top FOTS surface (layer thicknesses not to scale).

S2. Sample Preparation

The final fabricated samples were cleaned with acetone, ethanol and IPA, and rinsed with DI water. After solvent cleaning, the samples were placed in oxygen plasma for 5 minutes. Before every set of experiments, the samples were rinsed with DI water again. The drop contact angle on the fabricated surfaces is measured using a goniometer and images shown in Table S1. The water to be used in the experiments was degassed by boiling it for one hour using hot-plate, and subsequently cooled down to room temperature in a sealed bottle. The liquids (regular or degassed DI water) were passed through a filter with pore size of 220 nm to eliminate any particles or contaminants suspended in the liquid. A pool of water was formed in a 6 cm long and 1.4 cm inner diameter glass tube bonded on the SiO_2 or FOTS surface to achieve boiling. The laser beam illuminated the sample normally through an inverted optical microscope, and the laser power was increased incrementally until the bubble formed. After a stable bubble was formed, the first reading of the stage *z* position was taken; bubble diameter and bubble base readings were obtained from the calibrated optical images that are simultaneously recorded. The laser power was increased by 20 mW per reading and measurements were stopped immediately before the damage threshold for the sample was reached.

Table S1: Drop contact angle on surfaces used in the boiling experiments

Sample Surface	Drop Contact Angle	Experimental Image of Drop
Hydrophilic SiO_2	0°	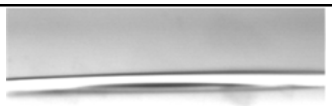
Normal SiO_2	$33.4^o \pm 2.7^o$	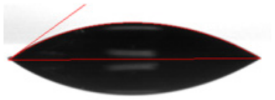
Trichlorosilane (FOTS)	$109.8^o \pm 2.9^o$	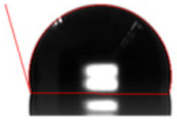

S2.1 Diffusion of Air in Degassed Water

In order to determine the duration during which the degassed water could remain degassed in the vicinity of the SiO_2 surface, the diffusion of air was simplified to a 1-D problem:

$$\frac{\partial C}{\partial t} = D\frac{\partial^2 C}{\partial x^2} \qquad \text{Eq. (1)}$$

where *C(x,t)* is concentration of air in water, *D* is diffusivity of air in water, and *t* is time. The following initial and boundary conditions were imposed: $(x,0) = 0$ and $C(0,t) = C_0$, where C_0 is the saturation concentration of air in the water at 101.3 kPa and 25 ^{o}C.

Solving Eq. (1), the concentration profile as a function of time is shown in Fig. S2 (Eq. 2). Thus, the length of the glass tube was chosen to be 6 cm to keep the water degassed for time scales much longer than the each experiment (approx. $t = 1$ h).

$$C(x,t) = C_0\text{erfc}(\frac{x}{\sqrt{4Dt}}) \qquad \text{Eq. (2)}$$

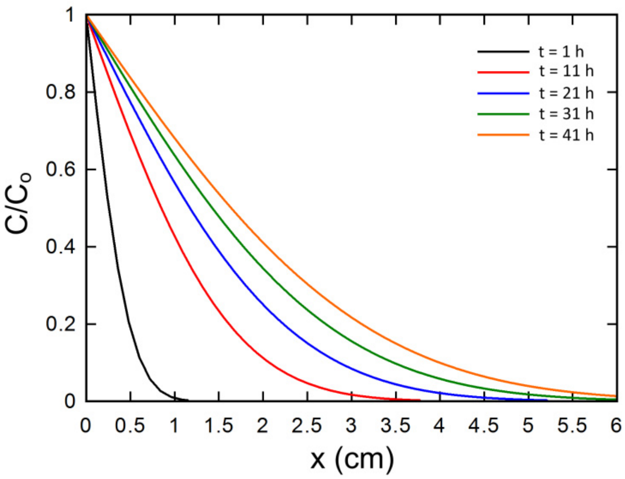

Figure S2: Diffusion profile of air in degassed water as a function of time to determine the height of glass tube.

S3. Experimental Setup

A Ti:Sapphire ultrafast laser was used to generate femtosecond laser pulses with an average power of 2.7 W at 800 nm (pulse length ≈ 120 fs, repetition rate = 80 MHz, center wavelength λ_0 = 800 nm). The laser pulses were then passed through a Second Harmonic Generation unit to generate 400 nm pulses with an average power of 450 mW. The average power of the laser illuminating the sample was controlled by using a continuously variable neutral density (ND) filter. The laser beam was then directed to one of the input ports of an inverted optical microscope. The laser beam was focused onto the sample using a 5× or 50× microscope objective. The laser pulses were partially absorbed by the thin metallic layers on the sample and created a stable and highly localized heating area corresponding to the beam size. A 6 cm long glass tube was mounted on the sample to hold the DI water. As the laser power was increased, a nucleation site on the sample surface was formed creating a stationary bubble inside the glass tube. The length of the tube was chosen to prevent diffusion of gasses back into the degassed water to the sample surface where the bubbles are generated. The bubbles were illuminated with a white light halogen lamp and imaged onto a CCD camera through the same microscope objective. To block the residual 400 nm laser beam reflected by the sample, a 425 nm long-pass filter was placed after the beam splitter. Various z-planes of the sample were focused using the motorized control for z-position of the objective (with a z-resolution of approx. 10 nm). The difference between two z-plane readings along with the bubble diameter measurements were used to calculate the bubble contact angle.

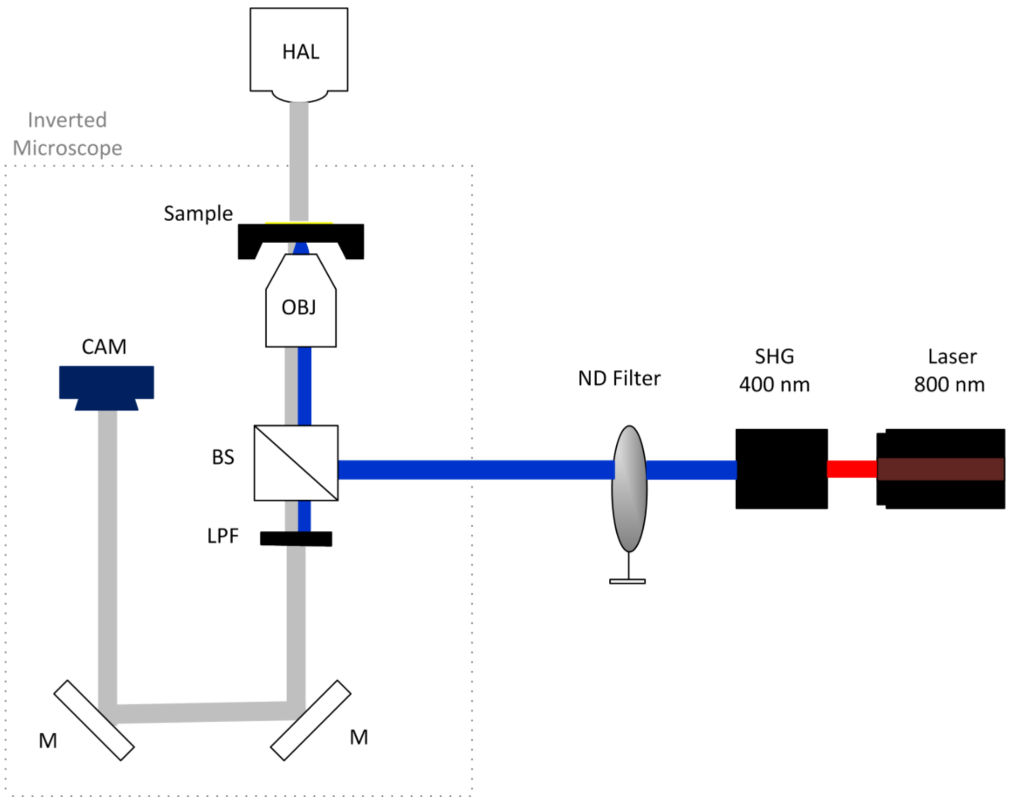

Figure S3: Schematic of the experiment setup for bubble contact angle measurement. SHG: Second Harmonic Generator, ND Filter: Continuously variable neutral density filter, HAL: white light halogen lamp for illumination, OBJ: 5× or 50× microscope objective, BS: beam splitter, LPF: 425 nm Long Pass Filter, M: mirror, CAM: 5 megapixel CCD camera.

In order to calculate the power absorbed (P_{ab}) by the sample, a power meter was placed in the path of incident light to measure the input power (P_{in}). Similarly, the reflected (P_{re}) and the transmitted powers (P_{tr}) were measured. The absorbed power was obtained using Eq. (3) from energy conservation to be $P_{ab} = 0.52P_{in}$.

$$P_{ab} = P_{in} - P_{re} - P_{tr} \qquad \text{Eq. (3)}$$

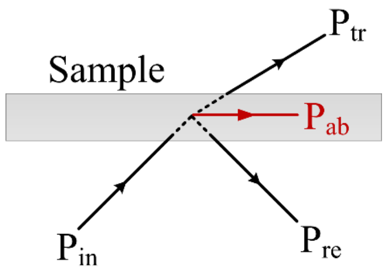

Figure S4: Schematic representing the incident laser on fabricated sample. The input laser power P_{in} is partially reflected P_{re} and transmitted P_{tr}; these three variables are experimentally measured to determine the absorbed laser power P_{ab} by the Au layer in the sample.

S4. Bubble Volume

In order to determine the volume of the bubble on the SiO_2 surface, it is divided into two parts (Fig. S5): top part (I) is a hemisphere while the lower part (II) is a partial parabola. Equations for the two sections can be determined by bubble base diameter D_{bb}, bubble diameter D_{bd} and corresponding height z of bubble middle plane:

$$y = \frac{1}{4z}x^2 - \frac{{D_{bb}}^2}{16z}, \qquad x \in \left(-\frac{D_{bd}}{2}, -\frac{D_{bb}}{2}\right) \cup \left(\frac{D_{bb}}{2}, \frac{D_{bd}}{2}\right) \qquad \text{Eq. (4)}$$

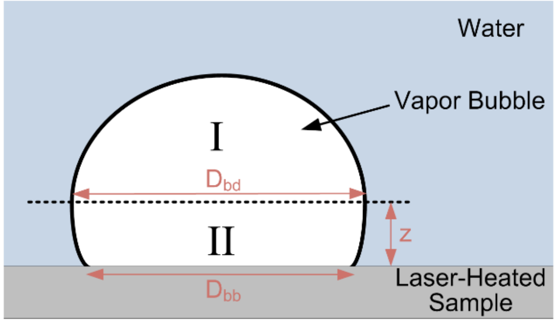

Figure S5: Schematic showing a vapor bubble formed on the laser-heated sample in a pool of water. The bubble is approximated as a combination of hemisphere top part (denoted as I) and parabolic lower part (denoted as II). Experimentally measured bubble mid-plane height (z), mid-plane diameter (D_{bd}) and bubble base diameter (D_{bb}) are used to estimate the bubble volume.

The volume of top part I is:

$$V_I = \frac{1}{12}\pi {D_{bd}}^3 \qquad \text{Eq. (5)}$$

For an infinite element in lower part II, the volume is $\pi x^2 \cdot dy$. Thus, the volume of part II is:

$$V_{II} = \int_0^z \pi(4zy + \frac{{D_{bb}}^2}{4})\, dy = \pi z(2z^2 + \frac{{D_{bb}}^2}{4}) \qquad \text{Eq. (6)}$$

Finally, the bubble volume is the sum of part I and part II:

$$V = \frac{1}{12}\pi {D_{bd}}^3 + \pi z(2z^2 + \frac{{D_{bb}}^2}{4}) \qquad \text{Eq. (7)}$$

S5. Finite-Element Numerical Simulations: *h* vs. *w* plot:

In the numerical simulations, the heat transferred through the evaporating region q was expressed as:

$$q = h \cdot \pi \left[\left({D_{bb}}/{2} + w \right)^2 - \left({D_{bb}}/{2} \right)^2 \right] \cdot \Delta T \quad \text{Eq. (8)}$$

where h is the heat transfer coefficient in the evaporating region, D_{bb} is the bubble base diameter, w is the width of the evaporating region, and ΔT is the temperature difference between the surface temperature in evaporating region to that of bulk liquid. Eq. (8) can be further simplified to:

$$q = h\pi(D_{bb}w + w^2)\Delta T \quad \text{Eq. (9)}$$

D_{bb} is in the order of 100 μm and w in the simulation is varied from 0.5 μm to 19.5 μm. Thus, the term including w^2 can be ignored making q highly dependent on the product of h and w. As the heat transfer q determines the evaporation rate and bubble volume growth rate, there exists an upper and a lower limit of the product of h and w, the region within which corresponds to a specified error between numerical simulations and experiments.

S6. Thermal Boundary Layer Thickness Estimation

COMSOL simulations are performed to determine the boundary layer thickness δ prior to bubble nucleation (similar to quenching heat flux) and the thickness varies between ~180-270 μm in the region above the heat source. The thermal boundary layer thickness is also estimated around the steady-state vapor bubble to be ~280 μm. Conduction heat transfer in water is assumed as the heat transfer mechanism in the thermal boundary layer.

Quenching heat flux: The domain for COMSOL simulations is described in Fig. S6-a, where the conditions prior to bubble nucleation are simulated. From Fig. 2e of manuscript, it is evident that a bubble does not form until the laser power exceeds ~100 mW. Thus, as the laser is turned on gradually from 0 mW, the laser power first heats up the water next to the surface (without bubble nucleation) forming a thermal boundary layer. This process is similar to that seen in traditional boiling methods when the bubble has departed from the surface and the heat flux at the surface is defined as the quenching heat flux which forms the thermal boundary layer. Simulations were performed for two laser powers: 50 mW and 100 mW, for the domain similar to experiments and that adapted in Fig. 4 of the manuscript. The thermal boundary layer thickness variation for these cases is shown in Fig. S6-b, as the temperature varies along the surface. Above the laser beam (heat source) area, the average thermal boundary layer thickness for 50 mW and 100 mW laser powers are found to be ~248 μm and ~195 μm, respectively.

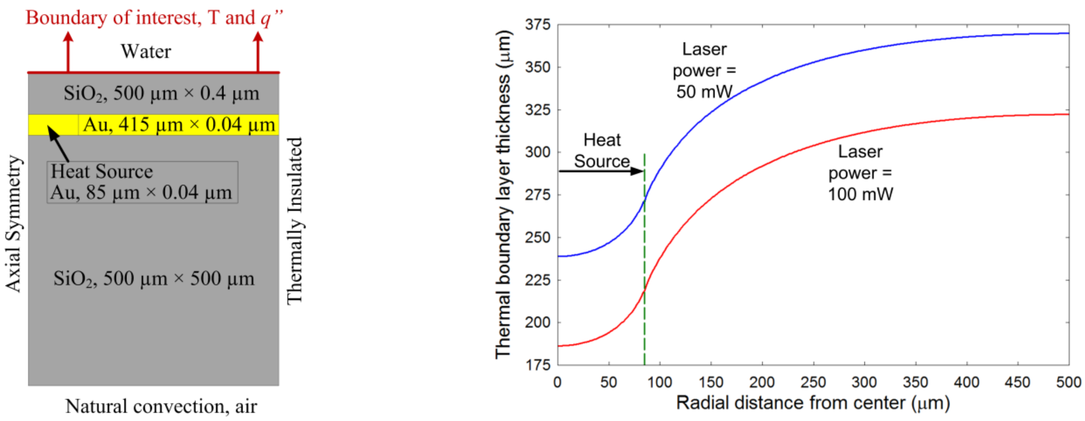

(a) (b)

Figure S6: (a) Schematic of the domain simulated using COMSOL simulations to determine the thermal boundary layer thickness prior to the nucleation of the bubble. (b) Thermal boundary layer thickness from the simulations for two laser powers of 50 mW and 100 mW.

Vapor bubble growth: The laser power q_{total} is dissipated by evaporation in evaporating region q_{ev} and natural convection outside the evaporating region q_{nc} (heat loss from underneath the sample is found to be negligible):

$$q_{total} = q_{ev} + q_{nc} \qquad \text{Eq. (10)}$$

Using data from Fig. 4e in the manuscript, we consider a case when h = 120 kW/m^{2}K and w = 10 μm, with D_{bb} = 133 μm. Our numerical simulation (Fig. 4 in manuscript) results in natural convection dissipating ≈ 55% of the laser power. The thermal boundary thickness in natural convection region can be obtained by:

$$q_{nc}" = k\frac{T_{wall} - T_{bulk}}{\delta} \qquad \text{Eq. (11)}$$

where the area to calculate natural convection heat flux is the annulus with outer radius of 1 mm and inner radius of 140 μm (taken from the simulation domain in Fig. 4d in manuscript), k is thermal conductivity of water, T_{wall} is from Fig. 4f in the manuscript, T_{bulk} is bulk liquid temperature at 25°C, and δ is thermal boundary thickness. From Eq. (11), the thermal boundary layer thickness is calculated to be ≈ 280 μm.

S7. Force Estimation on Complete Microlayer Wetted Bubble Base

Figure S7-a shows the main forces acting on the bubble when the entire base is wetted by the microlayer. The buoyancy force and capillary force (at the top curvature of bubble) aim to depart the bubble from the surface, while the reduced liquid pressure in microlayer (due to capillary and disjoining forces) want to keep the bubble attached on the surface. These forces are estimated below, which show that the sum of the forces that hold the bubble on the surface (1.14×10^{-5} N)

is greater than the sum of the forces that try to depart the bubble from the surface (5.16×10^{-6} N), thus preventing the bubble from departing even in the absence of a three-phase contact line.

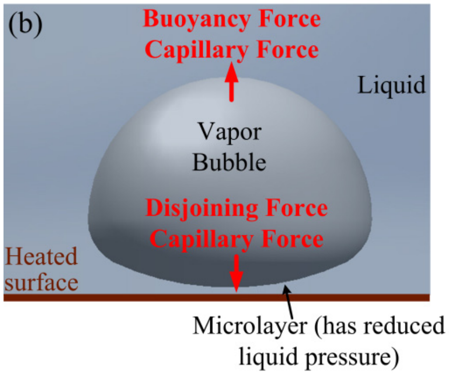

(a)

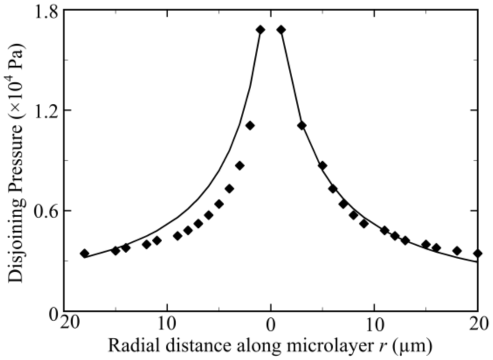

(b)

Figure S6: (a) Force balance on a bubble with completely wetting microlayer, and (b) radial variation of disjoining pressure in the microlayer based on conservative estimate.

Forces aiming to depart the bubble from the surface: The buoyancy force F_b can be estimated as: $F_b = \rho_l gV$, where ρ_l is the displaced liquid density, g is the gravitational acceleration, and V is the vapor volume of the bubble. From Fig. 3-b2 of manuscript, we estimate the bubble volume (partial sphere geometry) to be 3.30×10^{-13} m^3, thus resulting in $F_b = 3.23 \times 10^{-9}$ N. The pressure in liquid is lower than that inside the bubble, given by y $\Delta P = \frac{2\sigma}{R}$ where σ is the surface tension of liquid-vapor interface, and R is the radius of curvature of upper liquid-vapor interface. At the top of the bubble, the bubble radius is about 35 μm (Fig. 3-b3 in manuscript). The pressure difference between the vapor and bulk liquid is 4113 Pa. This pressure is acting on a radius equivalent to the bubble base microlayer radius of 20 μm (Fig. 3-b3 in manuscript). Hence, the force due to this pressure difference is 5.16×10^{-6} N. Thus, the sum of the forces that lift the bubble to depart it from the surface is ~5.16×10^{-6} N.

<u>Forces holding the bubble to the surface:</u> The curvature of the microlayer is obtained from fitting a parabolic curve to the microlayer profile using data from Fig. 3-b3 in manuscript, and is estimated to be 0.02 μm^{-1}. This curvature reduces the pressure in microlayer by ~ 2880 Pa. Taking the bubble base radius of 20 μm (Fig. 3-b3 in manuscript), the force due to this pressure difference is 3.62 × 10^{-6} N. A conservative estimation of disjoining pressure is performed by using data for non-polar liquid due to the lack of predictive models for water. The theoretical DLVO model has many unknown parameters making its use impractical. The data of carbon tetrachloride (CCl_4) on glass is used (Carey, V. P. Liquid-Vapor Phase-Change Phenomena, 2nd ed., Taylor & Francis, 2007). Since CCl_4 is non-polar while water is polar, the disjoining pressure estimated here is lower than the real case. The disjoining pressure P_d can be determined from $P_d=A\delta^{-B}$ where where A is a constant of 1.782 Pa•m^B and B = 0.6 for CCl_4; δ is the film thickness. Figure S7-b shows the disjoining pressure distribution in the microlayer. The average value can be obtained from Eq. (12) as 6184 Pa, resulting in a force (7.77 × 10^{-6} N). Thus, the sum of the forces that hold the bubble on the surface is 1.14 × 10^{-5} N.

$$P_{d,ave} = \frac{\int_1^{20} \frac{10^4}{(0.4497 + 0.1483r)} dr}{(20-1)} \qquad \text{Eq. (12)}$$

<u>Worst case Scenario:</u> In the calculation above, the microlayer thickness at the center is unknown and was assumed to be ~300 nm (which would lead to higher disjoining pressure), from which the outermost microlayer thickness was taken to be ~3 μm. Even if we assume the maximum film thickness which allows us to see fringes as 10 μm, the force due to the disjoining pressure will be 2.52 × 10^{-6} N. Thus, the sum of the forces (6.14 × 10^{-6} N) holding the bubble on the surface will still be greater than the sum of the forces (5.16 × 10^{-6} N) trying to depart the bubble from the surface. Further, P_d would be even greater for water thus further magnifying the forces holding the bubble onto the surface.

S8. Video Legends

Video 1: Steady state bubble formation on hydrophilic SiO_2 surface with degassed water. The bubble forms on the surface due to the incident laser and remains stable as the evaporation rate at the base of the bubble equals the condensation rate of vapor at the bubble's liquid-vapor interface. As the laser is blocked, the vapor within the bubble condenses causing the bubble to shrink and collapse.

Video 2: Steady state bubble formation on hydrophobic FOTS surface with degassed water due to incident laser. As expected, the bubble size is larger in size compared to the hydrophilic SiO_2 surface. The bubble achieves steady state as the evaporation rate at the base of the bubble equals the condensation rate of vapor at the bubble's liquid-vapor interface. The vapor within the bubble condenses as the laser is blocked, causing the bubble to shrink and collapse.

Video 3: Steady state bubble formation on hydrophilic SiO_2 surface with regular water containing dissolved air. The bubble size is much larger in size compared to the hydrophilic SiO_2 surface using degassed water. Further, the bubble keeps growing even at constant laser power as dissolved air is continuously released into the bubble along with evaporation of water. The vapor generation rate equals the condensation rate at the bubble's liquid-vapor interface; however, the air released into the bubble keeps accumulating causing the bubble to grow in size. As the laser is blocked, the vapor within the bubble condenses causing the bubble to shrink slightly. Now the bubble is comprised only of air and remains stable as the diffusion of air into the surrounding water (almost saturated with air) is a slow process.
